# Radiotherapy regimens for rectal cancer: long-term outcomes and health-related quality of life in the Stockholm III trial

**DOI:** 10.1093/bjsopen/zrab137

**Published:** 2022-01-18

**Authors:** Johan Erlandsson, Stina Fuentes, Calin Radu, Jan-Erik Frödin, Hemming Johansson, Yvonne Brandberg, Torbjörn Holm, Bengt Glimelius, Anna Martling

**Affiliations:** 1 Department of Molecular Medicine and Surgery, Karolinska Institutet, Stockholm, Sweden; 2 Department of Immunology, Genetics and Pathology, Experimental and Clinical Oncology, Uppsala University, Uppsala, Sweden; 3 Department of Oncology-Pathology, Karolinska Institutet, Stockholm, Sweden; 4 Department of Clinical Science and Education, Södersjukhuset, Karolinska Institutet, Stockholm, Sweden

## Abstract

**Background:**

The Stockholm III trial randomly assigned 840 patients to short-course radiotherapy of 5 × 5 Gy with surgery within 1 week (SRT), short-course radiotherapy of 5 × 5 Gy with surgery after 4–8 weeks (SRT-delay), or long-course radiotherapy of 25 × 2 Gy with surgery after 4–8 weeks (LRT-delay). This study details the long-term oncological outcomes and health-related quality of life (HRQoL).

**Methods:**

Patients with biopsy-proven resectable adenocarcinoma of the rectum were included. Primary outcome was time to local recurrence (LR), and secondary endpoints were distant metastases (DMs), overall survival (OS), recurrence-free survival (RFS), and HRQoL. Patients were analysed in a three-arm randomization and a short-course radiotherapy comparison.

**Results:**

From 1998 to 2013, 357, 355, and 128 patients were randomized to the SRT, SRT-delay, and LRT-delay groups respectively. Median follow-up time was 5.7 (range 5.3–7.6) years. Comparing patients in the three-arm randomization, the incidence of LR was three of 129 patients, four of 128, and seven of 128, and DM 31 of 129 patients, 38 of 128, and 38 of 128 in the SRT, SRT-delay, and LRT-delay groups respectively. In the short-course radiotherapy comparison, the incidence of LR was 11 of 357 patients and 13 of 355, and DM 88 of 357 patients and 82 of 355 in the SRT and SRT-delay groups respectively. No comparisons showed statistically significant differences. Median OS was 8.1 (range 6.9–11.2), 10.3 (range 8.2–12.8), and 10.5 (range 7.0–11.3) years after SRT, SRT-delay, and LRT-delay respectively. Median OS was 8.1 (range 7.2–10.0) years after SRT and 10.2 (range 8.5–11.7) years after SRT-delay. There were no statistically significant differences in HRQoL.

**Conclusion:**

After a follow-up of 5 years, delaying surgery for 4–8 weeks after radiotherapy treatment with 5 × 5 Gy was oncologically safe. Long-term HRQoL was similar among the treatment arms.

**Trial registration number:**

NTC00904813

## Teaser

Analysis of long-term follow-up after radiotherapy and surgery for rectal cancer showed that delaying surgery for 4–8 weeks after radiotherapy treatment with 5 × 5 Gy seems oncologically safe. HRQoL does not seem to differ between treatment arms with radiotherapy and immediate surgery, compared with delayed surgery.

## Introduction

Preoperative radiotherapy (RT) in rectal cancer is used to reduce local recurrences (LRs), with a demonstrable positive impact on overall survival (OS)^1^^,^^2^. Improved surgery, with the introduction of total mesorectal excision (TME), has improved outcomes and this has been enhanced by RT, reducing the rate of LRs by more than 50 per cent^3^^,^^4^.

Short-course RT (SRT) (5 × 5 Gy over 1 week), followed by surgery within 1 week, has been used in some European countries^5^^,^^6^. The alternative is to delay surgery for 4–8 weeks after SRT (SRT-delay) and this approach was included in the Stockholm III trial protocol due to local experiences where some tumours showed significant downsizing or even a complete response when surgery had been delayed inadvertently. This approach was predominantly used for patients who did not tolerate standard treatment with chemoradiotherapy (CRT) for locally advanced, non-resectable rectal cancers where downsizing/downstaging was necessary, described both from Sweden and UK^7–9^. A third option was long-course RT (LRT) with 2 Gy delivered in 25 fractions (LRT-delay). This was the standard preoperative treatment until three different trials showed that concomitant administration of a fluoropyrimidine to LRT, that is CRT, improved local control but not OS^10–12^.

**Fig. 1:**
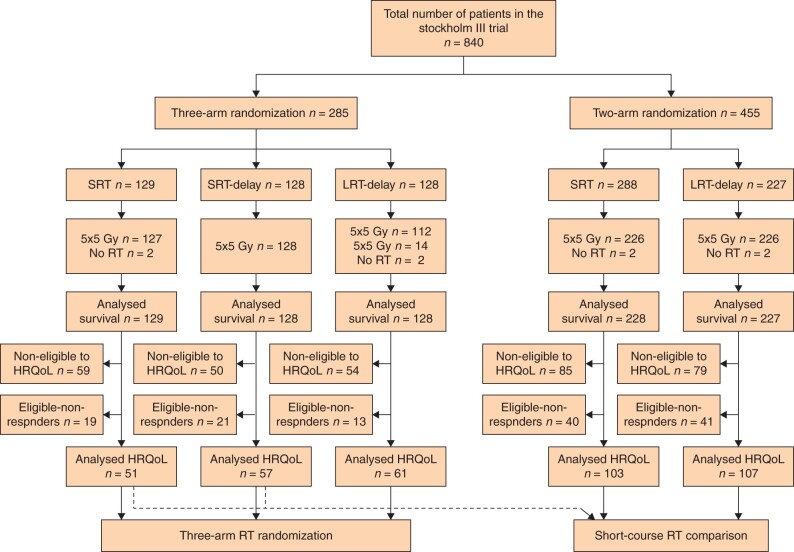
Flow chart Stockholm III trial SRT, short-course radiotherapy; LRT, long-course radiotherapy; RT, radiotherapy; HRQoL, health-related quality of life.

The Stockholm III trial randomly assigned patients with primarily resectable rectal cancer (with rigid sigmoidoscopy demonstrating a tumour ≤ 15 cm from the anal verge) to SRT, SRT-delay, or LRT-delay (*Fig. 1*). After a minimum follow-up of 2 years, oncological outcomes were similar in the three treatment groups, but with fewer postoperative complications in the groups with delayed surgery^13^.

The benefits of RT must be balanced against the risks of both early and long-term side-effects. Early toxicity is higher after prolonged RT, particularly if chemotherapy (CT) is added^5^^,^^14^. Although patients report more pain, fatigue, and insomnia during the first 6 months after RT, most patients return to their pretreatment levels within 2 years^15^. However, long-term effects on gastrointestinal, urogenital, and sexual function clearly affect health-related quality of life (HRQoL), even decades after initial treatment^16–18^.

The aim of this phase of the study was to analyse local and distant recurrences and long-term survival in the Stockholm III trial after a minimum follow-up of 5 years, and to analyse long-term HRQoL after a minimum follow-up of 3 years.

## Methods

The design and early results of the Stockholm III trial, which included patients enrolled from November 1998 to January 2013, have been presented previously^9^^,^^13^.

Patients with a biopsy-proven adenocarcinoma of the rectum, planned for a rectal resection, were included. Those with severe cardiovascular co-morbidities or previous RT to the pelvis were excluded.

The primary endpoint was time to LR, and other outcomes included distant metastases (DMs), OS, postoperative complications, and late morbidity. Early after initiation of the trial, an amendment to the protocol was proposed and endpoints regarding HRQoL and tumour regression were added. Tumour regression was analysed in 2019, with pathologists performing the reassessment blinded to treatment and previous staging^19^. Recruiting centres could choose to randomize patients to any of all three options, that is SRT, SRT-delay, or LRT-delay, or to apply a two-arm randomization between SRT and SRT-delay. Surgical options included anterior resection, abdominoperineal excision, or Hartmann’s procedure (all with the TME technique). All patients underwent surgery via an open approach. Patients were reported to the Swedish ColoRectal Cancer Registry (SCRCR), and reported data were used as the clinical reporting form. In the SCRCR, data are recorded prospectively by surgeons, pathologists, and oncologists. The registry has been validated several times and in 2018, it was shown to have a national coverage of more than 97 per cent^20^. Standard reporting intervals in the SCRCR is after surgery, at years 1, 3, and 5, or earlier if a recurrence is detected. In Sweden, the standard follow-up programme for rectal cancer is terminated if no events have occurred within 5 years after surgery. However, the SCRCR guideline states that any late recurrence detected after 5 years should be reported to the SCRCR. For patients in the trial, the participating centres were asked to verify that there were no recurrences in patients who did not have 5-year follow-up. Survival data in the SCRCR are linked to the Swedish Population Register and updated weekly when a patient has deceased. For recurrence and survival analyses, the last day of follow-up was set for 31 March 2018 when all patients had been followed up for at least 5 years after surgery.

### HRQoL

In 2004, all patients without LR or DM and with a minimum follow-up of 3 years were invited to participate in a questionnaire survey. Patient invitation was by mail, and reminders sent if patients had not replied within 2 weeks. For those who were eligible and accepted to participate, the European Organization for Research and Treatment of Cancer (EORTC) Quality of Life Core Questionnaire C30, version 3.0 (QLQ-C30) was sent out at one or two timepoints, before or after 6 years from the time of inclusion. For the analyses in this report, data from one questionnaire for each patient that was closest in time to 4–6 years were included in the analyses^21^^,^^22^.

The EORTC QLQ-C30 consists of 30 questions on global assessment, including two questions and five functional scales (physical, role, emotional, cognitive, and social) where high scores indicate high level of functioning, three symptom scales (fatigue, nausea and vomiting, and pain), and six single-symptom items (dyspnoea, insomnia, appetite loss, constipation, diarrhoea, and financial difficulties) where high scores indicate a high number of symptoms. Patients in the Stockholm III trial, as a whole group, were compared with EORTC QLQ-C30 data from a standard Swedish population matched for age and sex^23^.

Patients randomized to LRT-delay had tumours at a greater distance from the anal verge and consequently a higher frequency of anterior resections and less permanent stomas. Because of this and the permissive randomization protocol, patients randomized to SRT, SRT-delay, and LRT-delay in the three-arm comparison were analysed separately. Patients randomized to SRT and SRT-delay in both the three- and two-arm randomization were pooled and analysed in a short-course RT comparison.

### Statistical methods

Sample sizes in the randomization arms were determined based on power calculation regarding the primary outcome time to LR. Incidence data were based on previous studies at the time of study planning when LR frequency was estimated to be about 15 per cent. The trial was designed as a non-inferiority study and the experimental arm (SRT-delay) was deemed non-inferior if the upper limit of a one-sided 90 per cent confidence interval of a hazard ratio (HR) did not exceed 1.7 regarding the primary outcome. However, after initiation of the trial, it became clear that the LR rates were significantly lower than initially estimated and a new power calculation was done. It was concluded that with the current sample size, non-inferiority could be decided at an upper confidence interval limit of 6.5. This was accepted, and sample sizes were not changed. The present study was analysed on an intention-to-treat (ITT) basis, that is patients remained in the groups to which they were allocated, independent of the therapy received. Continuous variables were presented as interquartile range (i.q.r.) and compared with the Kruskal–Wallis test. Dichotomous variables were analysed with the chi-square test and Fisher’s exact test when appropriate. OS was calculated as time between the date of randomization and death. Recurrence-free survival (RFS) was calculated from the date of randomization to the first event of LR, DM, or death. Survival data were analysed with the Kaplan–Meier method. HRs were calculated by Cox regression, stratified according to participating centres. Data are presented with 95 per cent confidence intervals, except for LR which are presented with 90 per cent confidence intervals.

The EORTC QLQ-C30 questionnaires were processed according to the scoring manual^24^. The scale used ranged from 0 to 100 points, with higher scores representing better HRQoL on the functional scales and lower HRQoL on the symptom scales and single-item measures. A difference of 5–9 points on the 100-point scale was considered a ‘small’ clinical difference, 10–19 points a ‘moderate’ clinical difference, and ≥ 20 points a ‘large’ clinical difference^25^. The chi-square test was used to compare baseline characteristics for the whole study population with those of the population participating in the HRQoL analyses, and Fisher’s test was used when appropriate. The expected mean for each of the scale scores was calculated by use of the age distribution in the whole HRQoL group, together with age-specific mean reference scale scores from the Swedish population^23^.

Statistical significance level was set as *P* < 0.050. STATA version 14.2 (StataCorp, College Station, Texas, USA) and R version 5.1 (R Core Team, R Foundation for Statistical Computing, Vienna, Austria) were used for statistical calculations and plotting of graphs.

## Results

Baseline characteristics of 840 randomized patients in the trial are presented in *Table 1*, along with baseline characteristics of 379 patients included in the HRQoL analyses (*Table 2*).

**Table 1:** Baseline characteristics, type of surgery and postoperative stage of the patients in the Stockholm III trial, sorted by three-arm randomization or pooled short-course radiotherapy comparison

	Patients included in the Stockholm III trial (*n* = 840)				
	Three-arm randomization			Short-course radiotherapy comparison	
	SRT (n = 129)	SRT-delay (n = 128)	LRT-delay (n = 128)	SRT (n = 357)	SRT-delay (n = 355)
**Age (years)**	67 (35–86)	67 (41–85)	66 (40–85)	67 (35–89)	67 (40–88)
**Sex**					
Men	81 (62.8%)	79 (61.7%)	73 (57.0%)	218 (61.1%)	213 (60.0%)
Women	48 (37.2%)	49 (38.3%)	55 (43.0%)	139 (38.9%)	142 (40.0%)
**Height from anal verge (cm)**					
0–5	50 (38.8%)	57 (44.5%)	31 (24.2%)	128 (35.9%)	125 (35.2%)
6–10	49 (38.0%)	49 (38.3%)	60 (46.9%)	140 (39.2%)	144 (4.6%)
11–15	30 (23.3%)	21 (16.4%)	35 (27.3%)	88 (24.6%)	84 (23.7%)
**Type of surgery**					
AR	79 (61.2%)	68 (53.1%)	93 (72.7%)	218 (61.1%)	204 (57.5%)
APE	47 (36.4%)	53 (41.4%)	24 (18.8%)	122 (34.2%)	132 (37.2%)
Hartmann’s	3 (2.3%)	6 (4.7%)	8 (6.3%)	17 (4.8%)	18 (5.1%)
LE	0 (0%)	1 (0.8%)	1 (0.8%)	0 (0%)	1 (0.3%)
NR	0 (0%)	0 (0%)	2 (1.6%)	0 (0%)	0 (0%)
**ypStage**					
I^*^	38 (29.5%)	55 (43.0%)	37 (28.9%)	96 (26.9%)	138 (38.9%)
II	43 (33.3%)	31 (24.2%)	46 (35.9%)	118 (33.1%)	86 (24.2%)
III	48 (37.2%)	31 (24.2%)	37 (28.9%)	134 (37.5%)	107 (30.1%)
IV	0 (0%)	7 (5.5%)	5 (3.9%)	7 (2.0%)	13 (3.7%)
X	0 (0%)	3 (2.3%)	1 (0.8%)	1 (0.3%)	9 (2.5%)

Data are median (range) or *n* (%).

*Includes patients with complete pathological response. SRT, short-course radiotherapy of 5 × 5 Gy, with surgery within 1 week; SRT-delay, short-course radiotherapy of 5 × 5 Gy, with a delay of 4–8 weeks to surgery; LRT-delay, long-course radiotherapy of 25 × 2 Gy, with surgery after 4–8 weeks; AR, anterior resection; APE, abdominal perineal excision; LE, local excision; NR, no resection.

**Table 2:** Baseline characteristics, type of surgery, and postoperative stage of patients in HRQoL analyses, sorted by three-arm randomization and pooled short-course radiotherapy comparison

	Patients included in HRQoL analyses (*n* = 379)				
	Three-arm randomization			Short-course radiotherapy comparison	
	SRT (n = 51)	SRT-delay (n = 57)	LRT-delay (n = 61)	SRT (n = 154)	SRT-delay (n = 164)
**Age (years)**	64 (35–79)	63 (41–83)	65 (40–79)	65 (35–81)	64 (39–80)
**Sex**					
Men	33 (64.7%)	40 (70.2%)	38 (62.3%)	94 (61.0%)	97 (59.1%)
Women	18 (35.3%)	17 (29.8%)	23 (37.7%)	60 (39.0%)	67 (40.9%)
**Height from anal verge (cm)**					
0–5	20 (39.2%)	25 (43.9%)	18 (29.5%)	53 (34.4%)	55 (33.5%)
6–10	20 (39.2%)	20 (35.1%)	28 (45.9%)	60 (39.0%)	60 (36.9%)
11–15	11 (21.6%)	12 (21.1%)	15 (24.6%)	41 (26.6%)	49 (29.9%)
**Type of surgery**					
AR	33 (64.7%)	34 (59.6%)	41 (67.2%)	98 (63.6%)	105 (64.0%)
APE	17 (33.3%)	22 (38.6%)	15 (24.6%)	50 (32.5%)	56 (34.1%)
Hartmann's	1 (2.0%)	1 (1.8%)	3 (4.9%)	6 (3.9%)	3 (1.8%)
LE	0 (0%)	0 (0%)	0 (0%)	0 (0%)	0 (0%)
NR	0 (0%)	0 (0%)	2 (3.3%)	0 (0%)	0 (0%)
**yPStage**					
I*	28 (54.9%)	30 (52.6%)	23 (37.7%)	65 (42.2%)	74 (45.1%)
II	14 (27.5%)	15 (26.3%)	20 (32.8%)	49 (31.8%)	38 (23.2%)
III	9 (17.6%)	10 (17.5%)	17 (27.9%)	39 (25.3%)	44 (26.8%)
IV	0 (0%)	0 (0%)	0 (0%)	0 (0%)	1 (0.6%)
X	0 (0%)	2 (3.5%)	1 (1.6%)	1 (0.6%)	7 (4.3%)

Data are median (range) or *n* (%).

*Includes patients with complete pathological response. HRQoL, health-related quality of life; SRT, short-course radiotherapy of 5 × 5 Gy, with surgery within 1 week; SRT-delay, short-course radiotherapy of 5 × 5 Gy, with a delay of 4–8 weeks to surgery; LRT-delay, long-course radiotherapy of 25 × 2 Gy, with surgery after 4–8 weeks; AR, anterior resection; APE, abdominal perineal excision; LE, local excision; NR, no resection.

Seven patients did not receive any RT, and 14 patients allocated to receiving 25 × 2 Gy had 5 × 5 Gy. Median follow-up time for OS was 9.8 (i.q.r. 7.7–12.6) years. Median follow-up time for patients in the HRQoL analyses was 4.3 (i.q.r. 3.1–11.6) years.

### Three-arm randomization (SRT, SRT-delay, and LRT-delay)

**Fig. 2:**
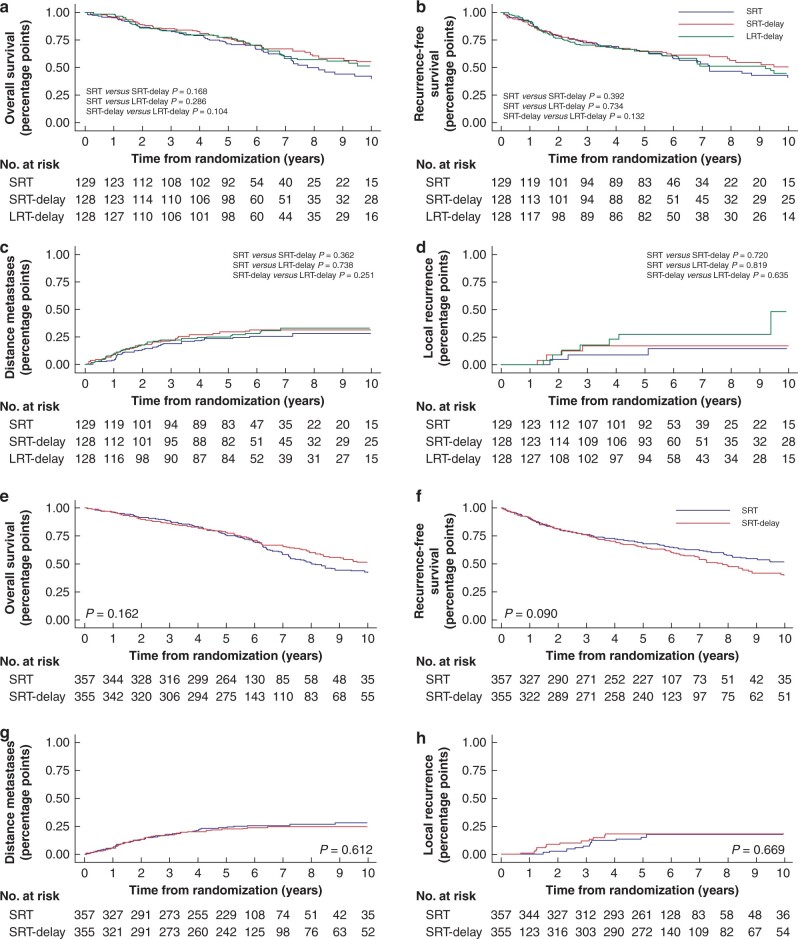
Oncological outcomes Three-arm randomization and short-course comparison.

There were no statistically significant differences among the treatment groups regarding LR, DM, RFS, or OS rates (*Fig. 2*). Oncological outcomes are presented in *Table 3*. Results on HRQoL in the three-arm randomization groups are presented in *Table 4*. Neither the functioning or symptom scales nor the single-item measures or global measure demonstrated any statistically significant differences among the groups. Baseline characteristics of eligible patients who did not respond to HRQoL questionnaires are presented in *Table 5*.

**Table 3:** Oncological outcomes and survival in the three-arm randomization and short-course radiotherapy comparison

Three-arm randomization					
	**SRT (*n* = 129)**	**SRT-delay (*n* = 128)**		**LRT-delay (*n* = 128)**	

Local recurrence	3 (2.3%)	4 (3.1%)		7 (5.5%)	
Distant metastases	31 (24.0%)	38 (29.7%)		38 (29.7%)	
Any death	65 (50.4%)	58 (45.3%)		56 (43.8%)	
Intercurrent death	37 (28.7%)	24 (18.8%)		23(18.0%)	

**Hazard ratio**	**SRT (reference)**	**SRT-delay**	***P***	**LRT-delay**	***P***

LR HR*	1.0	1.18 (0.33, 4.15)	0.830	2.23 (0.70, 7.14)	0.236
DM HR	1.0	1.47 (0.90, 2.42)	0.123	1.23 (0.76, 1.99)	0.390
OS HR	1.0	0.75 (0.51, 1.09)	0.137	0.88 (0.61, 1.29)	0.512
RFS HR	1.0	0.90 (0.61, 1.21)	0.589	0.99 (0.63, 1.30)	0.956

Short-course radiotherapy comparison			
	**SRT (*n* = 357)**	**SRT-delay (*n* = 355)**	

Local recurrence	11 (3.1%)	13 (3.7%)	
Distant metastases	88 (24.7%)	82 (23.1%)	
Any death	200 (56.0%)	211 (59.4%)	
Intercurrent death	84 (23.5%)	68 (19.2%)	

**Hazard ratio**	**SRT (reference)**	**SRT-delay**	***P***

LR HR^*^	1.0	1.31 (0.66,2.61)	0.518
DM HR	1.0	0.94 (0.69,1.27)	0.692
OS HR	1.0	0.84 (0.66,1.06)	0.146
RFS HR	1.0	0.85 (0.68,1.06)	0.144

Data are *n* (%) or hazard ratio (HR) with 95 per cent confidence intervals, unless otherwise specified.

*90 per cent confidence intervals. SRT, short-course radiotherapy and surgery within 1 week; SRT-delay, short-course radiotherapy, with surgery after 4–8 weeks; LRT-delay, long-course radiotherapy of 25 × 2 Gy, with surgery after 4–8 weeks; LR, local recurrence; DM, distant metastases; OS, overall survival; RFS, recurrence-free survival.

**Table 4:** EORTC QLQ-C30 three-arm randomization

EORTC QLQ-C30	SRT (*n* = 51)	SRT-delay (*n* = 57)	LRT-delay (*n* = 61)	*P*
	Mean scale score (s.d.)	Mean scale score (s.d.)	Mean scale score (s.d.)	
**Global health status**	**75 (19)**	**71(23)**	**71(21)**	**0.494**
**Functional scales**				
Physical functioning	86 (19)	85 (19)	84 (18)	0.851
Role functioning	81 (28)	82 (27)	79 (27)	0.868
Emotional functioning	86 (19)	82 (19)	84 (19)	0.525
Cognitive functioning	88 (16)	85 (17)	91 (17)	0.698
Social functioning	74 (28)	76 (29)	76 (29)	0.905
**Symptoms**				
Fatigue	21 (21)	24 (23)	24 (24)	0.626
Nausea and vomiting	6 (12)	6 (16)	2 (7)	0.091
Pain	19 (26)	19 (26)	17 (26)	0.812
**Single items**				
Dyspnoea	16 (22)	19 (24)	19 (25)	0.714
Insomnia	20 (25)	21 (29)	21 (29)	0.963
Appetite loss	5 (12)	6 (18)	10 (24)	0.397
Constipation	17 (27)	14 (24)	15 (29)	0.791
Diarrhoea	23 (30)	22 (31)	19 (30)	0.752
Financial difficulties	2 (11)	10 (23)	9 (20)	0.074

EORTC, European Organization for Research and Treatment of Cancer; QLQ-C30, Quality of Life Core Questionnaire C30, version 3.0; SRT, short-course radiotherapy and surgery within 1 week; SRT-delay, short-course radiotherapy, with surgery after 4–8 weeks; LRT-delay, long-course radiotherapy of 25 × 2 Gy, with surgery after 4–8 weeks.

**Table 5:** Baseline characteristics of eligible non-responders (alive and recurrence-free patients) who were invited to, but did not, participate in HRQoL analyses

	Three-arm randomization			Short-course radiotherapy comparison	
	SRT (*n* = 19)	SRT-delay (*n* = 21)	LRT-delay (*n* = 13)	SRT (*n* = 59)	SRT-delay (*n* = 62)
**Age (years)**	68 (56–77)	69 (60–78)	63 (48–85)	67 (49–81)	66 (42–86)
**Sex**					
Men	12 (63.2%)	7 (33.3%)	5 (38.5%)	34 (57.6%)	32 (51.6%)
Women	7 (36.8%)	14 (66.7%)	8 (61.5%)	25 (42.4%)	30 (48.4%)
**Height from anal verge (cm)**					
0–5	6 (31.6%)	10 (47.6%)	5 (38.5%)	22 (37.3%)	19 (30.6%)
6–10	8 (42.1%)	9 (42.9%)	8 (61.5%)	21 (35.6%)	31 (50.0%)
11–15	5 (26.3%)	2 (9.5%)	0 (0%)	16 (27.1%)	12 (19.4%)
**Type of surgery**					
AR	12 (63.2%)	10 (47.6%)	9 (69.2%)	38 (64.4%)	36 (58.1%)
APE	6 (31.6%)	10 (47.6%)	4 (30.8%)	20 (34%)	24 (38.7%)
Hartmann’s	1 (5.3%)	1 (4.8%)	0 (0%)	1 (21.7%)	2 (3.2%)
LE	0 (0%)	0 (0%)	0 (0%)	0 (0%)	0 (0%)
NR	0 (0%)	0 (0%)	0 (0%)	0 (0%)	0 (0%)
**YpStage**					
I*	3 (15.8%)	11 (52.4%)	7 (53.9%)	12 (20.3%)	27 (43.5%)
II	7 (36.8%)	6 (28.6%)	5 (38.5%)	24 (40.7%)	17 (27.4%)
III	9 (47.4%)	3 (14.3%)	1 (7.7%)	23 (39.0%)	17 (27.4%)
IV	0 (0%)	0 (0%)	0 (0%)	0 (0%)	0 (0%)
X	0 (0%)	1 (4.8%)	0 (0%)	0 (0%)	1 (1.6%)

Data are median (range) or *n* (%). *Includes patients with complete response. HRQoL, health-related quality of life; SRT, short-course radiotherapy of 5 × 5 Gy, with surgery within 1 week; SRT-delay, short-course radiotherapy of 5 × 5 Gy, with a delay of 4–8 weeks to surgery; LRT-delay, long-course radiotherapy of 25 × 2 Gy, with surgery after 4–8 weeks; AR, anterior resection; APE, abdominal perineal excision; LE, local excision; NR, no resection.

### Short-course RT comparison (SRT and SRT-delay)

There were no statistically significant differences between the two groups regarding LR, DM, RFS or OS rates (*Fig. 2*). HRs from Cox regression analysis are presented in *Table 3*.

Overall, 72 per cent of eligible patients completed the EORTC QLQ-C30 questionnaire. No statistically significant differences were found between the SRT and SRT-delay groups (data not shown).

### HRQoL comparison between recurrence-free rectal cancer patients and a Swedish reference population

**Fig. 3:**
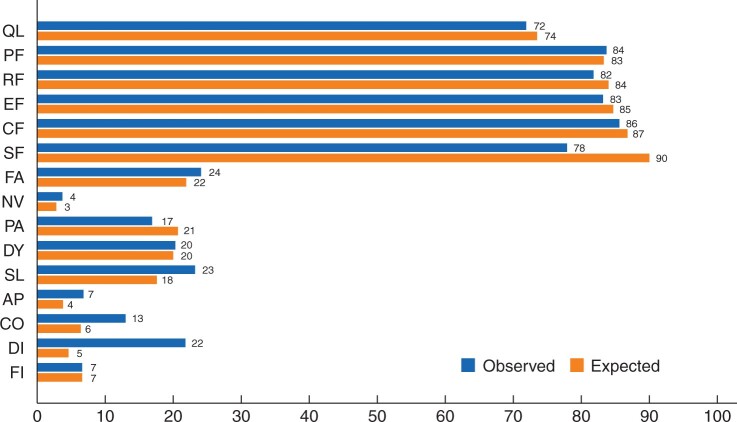
Mean values for EORTC QLQ-C30 subscales for the study sample (observed) and the Swedish reference population (expected) Expected scale scores are calculated by indirect standardization with age- and sex-specific scores from the Swedish population. EORTC, European Organization for Research and Treatment of Cancer; QLQ-C30, Quality of Life Core Questionnaire C30, version 3.0; QL, global quality of life; PF, physical functioning; RF, role functioning; EF, emotional functioning; CF, cognitive functioning; SF, social functioning; FA, fatigue; NV, nausea and vomiting; PA, pain; DY, dyspnoea; SL, insomnia; AP, appetite loss; CO, constipation; DI, diarrhoea; FI, financial difficulties.

Comparison of results from 379 study participants completing the EORTC QLQ-C30 questionnaire *versus* a reference population showed clinical differences in social functioning (‘moderate’), sleep disturbances (‘small’), and diarrhoea (‘moderate’), better functioning, and lower levels of symptoms in the reference population. None of the other scales showed clinical differences between the two populations (*Fig. 3*).

In the trial, data on CT received were not validated. Medical oncological treatment was not reported to the SCRCR before 2007. In the period from 2007 to 2013, 489 patients were included in the trial and 72 patients (14.7 per cent) received postoperative CT, including two patients with ypStage I, six patients with ypStage II, and 64 patients with ypStage III^13^.

## Discussion

Delaying surgery after preoperative treatment in rectal cancer has some major advantages: time for patient optimization, possibility of a complete clinical response, and lower risk of postoperative complications. However, it may delay the start of adjuvant chemotherapy or increase the risk of tumour progression, with a potentially worse oncological outcome. In this long-term follow-up of the Stockholm III trial, no statistically significant differences in oncological outcomes regarding LR, DM, RFS, OS, or HRQoL were found on comparison of the SRT, SRT-delay, and LRT-delay groups. Delaying surgery after short-course RT seems safe, at least when use of postoperative CT is limited, as it was in Sweden during the trial period.

The main advantage of this study is the randomized patient cohort with minimal differences in patient characteristics. All patients were followed up in the SCRCR, and data were validated in the patients’ medical charts. However, one possible risk is that late recurrences after 5 years were not reported to the SCRCR, especially if the recurrence was not diagnosed at a surgical or oncological department. This could impact the absolute number of recurrences; however, it is unlikely that there would be a difference in registry reporting, depending on the allocated treatment. In the present report, about 7 per cent of local or distant recurrences were diagnosed more than 5 years from randomization. Most recurrences are diagnosed within the first 2 years after surgery, although late recurrences can be seen after rectal cancer surgery^26^^,^^27^. Furthermore, the cumulative incidences of LR and DM are in line with what is expected considering the postoperative tumour stage.

An obvious limitation is the long inclusion period. Surgical treatment, preoperative staging, and postoperative care continually evolve^28^. The impact of these improvements on the different treatment strategies is unknown. In Sweden, initial treatment in rectal cancer is recommended at a multidisciplinary conference where MRI is the standard imaging for local tumour staging. However, data on the preoperative local tumour stage were only available in the SCRCR from 2007 and could not be analysed in this study. Treatment guidelines during the inclusion period emphasized the importance of categorizing tumours into three different groups, according to the ‘good, bad, or ugly’ concept^29^. Most patients included in the Stockholm III trial had tumours classified as ‘bad or intermediate risk’, although patients with stage I disease were included early in the trial. Since 2003 when MRI was implemented for rectal cancer staging, with a more precise preoperative staging, only patients with stage II and III disease were included. Patients with more advanced or ‘ugly’ tumours usually received CRT outside the Stockholm III trial^30^.

As improved surgical techniques and selective neoadjuvant treatments have reduced LR rates, the present focus on rectal cancer recurrence should be on DM. In the present study, no statistically significant differences in the rates of DM could be found between the arms, neither in the ITT analyses nor when comparing the as-treated groups (data not shown). Other trials comparing different RT regimens have found similar results. Local tumour treatment, including different overall treatment times (OTTs), does not seem to affect the rate of DM, at least not at a group level^31–34^. In addition, there were no differences in survival among the treatment arms in the three-arm randomization nor in comparing time to surgery after SRT.

The potential downside of delaying surgery after preoperative RT is prolongation of OTT. RT toxicity can be an issue and 6 per cent of patients in the arms with a delay to surgery required in-hospital care^13^. In patients with no or minor tumour regression following radiation, the prognosis is inferior, compared with those achieving an excellent response^35^. Whether 4–8 weeks of delay to surgery matters with respect to recurrence or survival in patients with non-responding tumours is unknown. The optimal waiting time before clinical and radiological evaluation after RT is not clear. The first signs of tumour regression following SRT can be found after an OTT of 10 days, but full regression effect may take several weeks to months^31^^,^^36–38^.

Tumour repopulation using [18F]fluorodeoxyglucose PET (FDG-PET) can be seen after 6–12 weeks in about half of the population treated with CRT^39^. The beneficial effect of adjuvant CT in patients who have received preoperative treatment remains controversial^40^; however, if postoperative CT is indicated, a prolongation of the time to start what may negatively influence outcome^41^. The RAPIDO trial included patients with locally advanced rectal cancer treated with SRT followed by CT for 4–5 months—this was well tolerated and reduced DM rates, compared with standard treatment with conventional CRT^42^.

In this study, there were no significant differences among the treatment arms in overall HRQoL after a minimum follow-up of 3 years. However, patients in the Stockholm III trial overall had worse scores related to diarrhoea and social functioning, compared with the reference population. Worse bowel function would be anticipated in a patient group that have been treated for rectal cancer.

In the present trial, it was not possible to detect any differences in age or other characteristics between responders and eligible non-responders.

There are limitations associated with qualitative research relating to HRQoL, especially if response rates differ among patients who are the most symptomatic compared with those satisfied with their quality of life and function. There could be inherent bias from patients who have been cured from cancer being thankful, thus impacting how they respond to the questions. Responding to a questionnaire at home may differ from providing responses in a clinical setting with a healthcare professional.

The present results have provided some reassurance for patients requiring rectal cancer treatment during the coronavirus disease (Covid-19) pandemic. A prolonged time between RT and surgery seems acceptable when good response to preoperative treatment has been demonstrated. SRT-delay has therefore been recommended, instead of CRT, to decrease the number of fractions, and thus to reduce hospital visits, in addition to the beneficial effect on the risk of developing postoperative complications^43^.

## Funding

This study was funded by the Swedish Research Council, Swedish Cancer Society and Stockholm Cancer Society, and the Stockholm County Council and Karolinska Institutet through regional agreement on medical training and clinical research.

*Disclosure*. The authors declare no conflict of interest.

## Data availability

The data underlying this article are available on request.
